# Molecular consequences of SOD2 expression in epigenetically silenced pancreatic carcinoma cell lines

**DOI:** 10.1038/sj.bjc.6604000

**Published:** 2007-09-25

**Authors:** E M Hurt, S B Thomas, B Peng, W L Farrar

**Affiliations:** 1 Cancer Stem Cell Section, Laboratory of Cancer Prevention, Center for Cancer Research, National Cancer Institute at Frederick, National Institutes of Health, Frederick, MD 21702, USA; 2 Basic Research Program, SAIC-Frederick Inc., National Cancer Institute at Frederick, Frederick, MD 21702, USA; 3 School of Dental Science, University of Melbourne, Melbourne, Victoria, Australia

**Keywords:** superoxide dismutase 2, pancreatic neoplasms, proteomics, genomics, epigenetic process

## Abstract

Manganese superoxide dismutase (SOD2) is an enzyme that catalyses the dismutation of superoxide in the mitochondria, leading to reduced levels of reactive oxygen species. Reduced expression levels of SOD2 have been shown to result in increased DNA damage and *sod2* heterozygous mice have increased incidences of cancer. It has also been shown that SOD2 expression is lost in pancreatic cell lines, with reintroduction of SOD2 resulting in decreased rate of proliferation. The mechanism of decreased SOD2 expression in pancreatic carcinoma has not been previously determined. We demonstrate, through sodium bisulphite sequencing, that the *sod2* locus is methylated in some pancreatic cell lines leading to a corresponding decrease in SOD2 expression. Methylation can be reversed by treatment with zebularine, a methyltransferase inhibitor, resulting in restored SOD2 expression. Furthermore, we demonstrate that sensitivity of pancreatic carcinoma cell lines to 2-methoxyestradiol correlates with SOD2 expression and SOD2 modulation can alter the sensitivity of these cells. Using both genomics and proteomics, we also identify molecular consequences of SOD2 expression in MIA-PaCa2 cells, including dephosphorylation of VEGFR2 and the identification of both SOD2-regulated genes and transcription factors with altered binding activity in response to SOD2 expression.

Free radicals are, arguably, considered among the most potent, ubiquitous, endogenous mutagens generated by normal physiological processes (Hussain *et al*, 2003). Among various molecular forms of free radicals, reactive oxygen species (ROS) have been the most diligently studied. Generated by all cells as a by-product of oxidative metabolism and inflammatory processes, ROS are indicted in inducing cellular aging (Melov, 2000), cancer, and a variety of other pathophysiological conditions. Alterations in macromolecular functions are understandable in view that ROS may target DNA, proteins, RNA, and lipids. Due to the deleterious potential of free radicals, mechanisms have evolved to protect cells from ROS, including antioxidant scavengers, enzymes, and repair mechanisms.

Cancer, in particular, arises from a combination of potential genetic mutations, and possibly epigenetic changes, altering the cells’ normal proliferative and apoptotic programming. Within the last few years, changes in expression patterns of enzymes that stand as guardians for ROS have been implicated in the possible progression or initiation of some cancers. Considered among the earliest pre-neoplastic changes in prostate cancer is the epigenetic silencing by promoter CpG hypermethylation of the glutathione *S*-transferase *π* gene (Lin *et al*, 2001; Nelson *et al*, 2001). Thus, speculating that a reduction in the ability to manage ROS leads to further mutation and advancement of the neoplastic process.

Among the enzymes produced by cells that manage ROS, manganese superoxide dismutase (SOD2), the form of SOD found in mitochondria, has received considerable attention with regard to cancer (Liu *et al*, 2004). The main function of SOD2 is to convert superoxide anion into hydrogen peroxide (H_2_O_2_), which is subsequently converted to water by catalases. Decreased expression of SOD2 has been noted, initially, in pancreatic carcinoma cells lines (Cullen *et al*, 2003). More recently, reduced expression of SOD2 has been seen in multiple myeloma cells (Hodge *et al*, 2005b), androgen-independent prostate cancer (Best *et al*, 2005; Venkataraman *et al*, 2005), and invasive breast carcinoma (Soini *et al*, 2001). Overexpression of SOD2 in deficient cell lines has reduced the proliferation of pancreatic carcinoma cells (Weydert *et al*, 2003; Ough *et al*, 2004), multiple myeloma cells (Hodge *et al*, 2005b), glioma cells (Zhong *et al*, 1997), squamous oral carcinoma cells (Liu *et al*, 1997), and prostate carcinoma cells (Li *et al*, 1998b; Zhong *et al*, 2004; Venkataraman *et al*, 2005). Transgenic overexpression of SOD2 also results in increased resistance to chemical carcinogenesis as compared to wild-type mice (Zhao *et al*, 2001). While homozygous *sod2* knockout mice die within weeks, heterozygous +/− mice have accelerated tumour development, particularly lymphomas (Van Remmen *et al*, 2003). Taken together, these data have raised the compelling issue that, in addition to the effects of SOD2 as a guardian against ROS, SOD2 may phenotypically act as a tumour suppressor (Cullen *et al*, 2003).

While the phenotypic effects of diminished and/or overexpressed SOD2 have been reported for various tumour cell lines, the explanation for reduced expression has only recently emerged. Polymorphisms of the *sod2* promoter have been suggested as one possible mechanism for reduced expression of SOD2 in certain cell lines (Xu *et al*, 1999). More recently, we have found that the *sod2* promoter is methylated in some multiple myeloma cells and diminished expression can be reversed by the methyltransferase inhibitor zebularine (Hodge *et al*, 2005a), suggesting that repression of SOD2 may occur at the level of epigenetic regulation. In addition to understanding the relative mechanisms of SOD2 expression levels in cancer cells, little is known about the consequences of reduced SOD2 on the signal transduction and transcriptional processes that control cancer cell viability and growth. Some data have emerged suggesting that levels of SOD2 have effects on AP-1 and NF-*κ*B activities, in which overexpression experiments diminish the activities of these two important transcription factors (Kiningham and St Clair, 1997; Li *et al*, 1998a).

Here, using a series of pancreatic carcinoma cell lines, we show that the relative expression patterns of SOD2 are inversely related to the methylation density status of the *sod2* promoter. Hypermethylation of CpG sites was rapidly reversed by the methyltransferase inhibitor zebularine, restoring SOD2 levels. The epigenetic silencing of *sod2* revealed a particular Achille's heel of pancreatic carcinoma cells with low SOD2, making the cells particularly susceptible to the apoptotic effects of 2-methoxyestradiol (2ME2), an oxidative burst agent.

To examine the more global consequences of SOD2 expression on the cellular processes of pancreatic carcinoma, we have employed a gestalt approach of examining how SOD2 affects vital processes of signal-transduction proteins, transcription factor activation, and gene regulation. Using reverse-phase antibody arrays for signal proteins, transcription factor arrays and gene expression microarrays, we have identified some of the major aspects in which SOD2 governs signal transduction and gene expression in pancreatic carcinoma cells.

## MATERIALS AND METHODS

### Genomic DNA isolation, bisulphite modification, and PCR amplification

Genomic DNA was isolated using Qiagen's DNA isolation kit (Qiagen). DNA was *Eco*RI digested, denatured with 0.3 M NaOH for 15 min at 37°C, and modified with 3.1 M sodium bisulphite and 0.5 mM hydroquinone. Modified DNA was purified using the QIAquick DNA extraction kit (Qiagen, Valencia, CA, USA) and treated with 0.3 M NaOH for 15 min at 37°C to complete the modification. Nested PCR was used to amplify the modified DNA fragments. The first set of primers for the *sod2* gene promoter region (Genbank accession no. L34157) was 5′-GtAtttTtAGGGG[C/t]GGAt[C/t]GGAGGtAGGGtTT-3′ (sense, nt 1896–1927) and 5′-CCAaaCCC[a/G]aTaC[a/G]aCCACTaTC[a/G]CCATTaC-3′ (anti-sense, nt 2520–2490), and the second set of primers was 5′-GGGt[C/t]GTAttAAtTttA[C/t]GGGGGtAGGGGt-3′ (sense, nt 1929–1958) and 5′-AaCCCCTTaCCCCTTaaaaC[a/G]TaACC[a/G]aaTCCC-3′ (anti-sense, nt 2468–2436), where lower case letters represent bisulphite-converted nucleotides. PCR cycles were as follows: 95°C for 30 s, followed by 35 cycles at 95°C for 30 s, 55°C for 30 s, 72°C for 2 min, and final extension at 72°C for 10 min. The amplified DNA fragment of expected size was cloned into pCR4-TOPO TA cloning vector (Invitrogen, Carlsbad, CA, USA). Ten individual clones for each cell line were sequenced.

### Construction of SOD2 expression and shRNA retroviruses

Adenoviral SOD2 construct (kind gift of JJ Cullen, University of Iowa) was *Eco*RI digested and *sod2* cDNA was cloned into the retroviral vector pLZRS-BMN-eGFP containing an IRES and eGFP cDNA. For overexpression experiments, the control was the empty eGFP vector. The oligos used for shRNA knockdown of *sod2* are as follows: 5′-GATCCCGGGGTTGGCTTGGTTTCAATATTCAAGAGATATTGAAACCAAGCCAACCCCTTTTTA-3′ and 5′-AGCTTAAAAAGGGGTTGGCTTGGTTTCAATATCTCTTGAATATTGAAACCAAGCCAACCCCGG-3′. The oligos were annealed and cloned into the *Bgl*II/*Hin*dIII sites of pRetroSuper (Brummelkamp *et al*, 2002).

### Generation of stable cell lines with altered SOD2 expression levels

A bicistronic retrovirus carrying *sod2* cDNA and eGFP or an empty vector (control) was used to create the overexpression cell lines. For knockdown of SOD2, the shSOD2-pRetroSuper or empty pRetroSuper was used to infect pancreatic cell lines. In both instances, the Phoenix Amphotropic cells (kind gift of Gary Nolan, Stanford University) were transfected using FuGENE 6 (Roche Diagnostics, Indianapolis, IN, USA), according to manufacturer's instructions. Two days following transfection, 3 ml of viral supernatant was used to infect 1–3 × 10^6^ pancreatic cells. For overexpression of SOD2, cells were flow sorted for high GFP expression, whereas for shSOD2 and control cells were selected with 1 *μ*g ml^−1^ puromycin 48 h post infection. Expression of SOD2 was determined by western blot (anti-SOD2, ab16954; Abcam, Cambridge, MA, USA).

### Determination of superoxide dismutase activity in cell lines

Superoxide dismutase activity was measured using Calbiochem's Superoxide Dismutase Assay Kit II according to manufacturer's directions. Briefly, 2 × 10^6^ cells were sonicated in 20 mM HEPES buffer containing 1 mM EGTA, 210 mM mannitol, and 70 mM sucrose.

Ten microlitres of lysate was mixed with 200 *μ*l of radical detector (tetrazolium salt) containing 1 mM potassium cyanide to inhibit Cu/Zn-SOD and 20 *μ*l xanthine oxidase. The reaction was incubated at room temperature for 20 min and read in a Genios (Tecan, Palm Springs, CA, USA) plate reader at 450 nm. One unit of SOD activity is defined as the amount of enzyme needed to exhibit 50% dismutation of the superoxide radical.

### Measurement of proliferation by MTT assay

Cells were seeded in triplicate at 2 × 10^4^ per well in a 96-well plate. The 3-(4,5-dimethylthiazol-2-yl)-2,5-diphenyltetrazolium bromide (MTT) assay was performed in accordance with the manufacturer's instructions (Chemicon, Temecula, CA, USA). Briefly, following addition of MTT, the cells were incubated at 37°C for 2 h. The precipitate was resuspended in isopropanol containing 0.04 N hydrochloric acid and read immediately using a Genios (Tecan) plate reader at 570 nm.

### 2-Methoxyestradiol treatment and comet assay

One million cells were treated with 3 μM 2ME2 for 48 h. DNA damage was assessed using the CometAssay kit (Trevigen, Gaithersburg, MD, USA). Briefly, 500–1000 cells were embedded in 1.0% LMAagrose on a Cometslide and lysed (2.5 M sodium chloride, 100 mM EDTA, pH 10, 1 mM Tris base, 1% sodium lauryl sarcosinate, and 1% Triton X-100). The slides were then electrophoresed in alkaline electrophoresis solution (300 mM NaOH and 1 mM EDTA) for 30 min at 300 mA. Cells were stained with SYBR green and viewed by fluorescence microscopy. The comet tail moment for 50 nuclei per treatment was measured using ImageJ software (http://rsb.info.nih.gov/nih-image).

### Oligonucleotide expression microarrays

Oligonucleotide microarrays were prepared by the National Cancer Institute's microarray core facility, using Operon's Human version 3.0 oligo set (Operon Biotechnologies, Huntsville, AL, USA) printed on UltraGAPS slides (Corning, Corning, NY, USA). This set contains 34 580 oligos, representing approximately 25 000 unique genes. Total RNA (20 *μ*g) was labelled in a reverse transcription (RT) reaction with an oligo-dT primer and either Cy3-dUTP or Cy5-dUTP. Following the RT reaction, the RNA was hydrolysed by incubation with NaOH. Probes were purified with a Microcon YM-30 column (Millipore, Billerica, MA, USA). Microarrays were prehybridised in 5 × SSC, 0.1% SDS, and 1% BSA for 1 h at 42°C, washed in distilled water and then in 100% ethanol, and air dried. Hybridisation of mixed Cy-3- and Cy-5-labelled probes was performed in 50% formamide, 10 × SSC, and 0.2% SDS overnight at 42°C. After hybridisation, slides were washed in decreasing concentrations of SSC (2 × SSC+0.1% SDS, 1 × SSC, 0.2 × SSC). Arrays were scanned in a GenePix 4000B scanner and analysed using GenePix Pro (Molecular Devices, Sunnyvale, CA, USA). Data analysis was preformed using Cluster and TreeView, offered by Michael B Eisen as freeware (http://rana.lbl.gov/EisenSoftware.htm).

### Reverse-phase protein arrays

Protein lysates were prepared for printing on reverse-phase protein arrays (RPAs) by lysis of 10 000 cells in T-PER (Pierce, Rockford, IL, USA). Five two-fold serial dilutions were made from each lysate. RPAs were printed as previously described (Paweletz *et al*, 2001). Briefly, arrays were printed on nitrocellulose-coated glass slides (FAST Slides, Whatman, Florham Park, NJ, USA) using a GMS 417 arrayer (Affymetrix, Santa Clara, CA, USA). Each array was incubated with primary antibody and detected using the catalysed signal amplification system (Dako, Carpinteria, CA, USA) and an Autostainer Universal Staining System (Dako). Slides were blocked with I-block (Tropix, Bedford, MA, USA), incubated with primary and secondary antibodies, streptavidin–biotin complex, biotinyl tyramide, streptavidin-peroxidase, and developed with 3,3′-diaminobenzidine tetrahydrochloride chromogen with washing between all steps. Total protein was determined by staining arrays with AuroDye Forte (GE Healthcare, Piscataway, NJ, USA). The arrays were scanned with an Epson flat-bed scanner (Epson, Long Beach, CA, USA) and quantitated using MicroVigene with the dilution curve module (ViGene Tech, Carlisle, MA, USA). For each sample, the slope of the regression line best fitting the linear range of the dilution curve was used to determine relative protein expression. All experimental values were normalised to total protein.

### DNA/protein arrays

Panomic's DNA/protein Combo arrays containing 345 consensus binding sites were hybridised according to manufacturer's instructions. Briefly, nuclear protein was extracted using Panomic's Nuclear Protein extraction kit following manufacturer's instructions. Fifteen micrograms of each protein lysate was used in the binding reaction. Following clean-up, the probe was hybridised at 45°C overnight. Data were quantitated using ImageQuant (Amersham Biosciences), and a binding site was considered to be regulated only if it showed reciprocal regulation in the overexpression and the shRNA cell lines.

## RESULTS

### *Sod2* is methylated in some pancreatic cell lines leading to decreased expression

**Figure 1:**
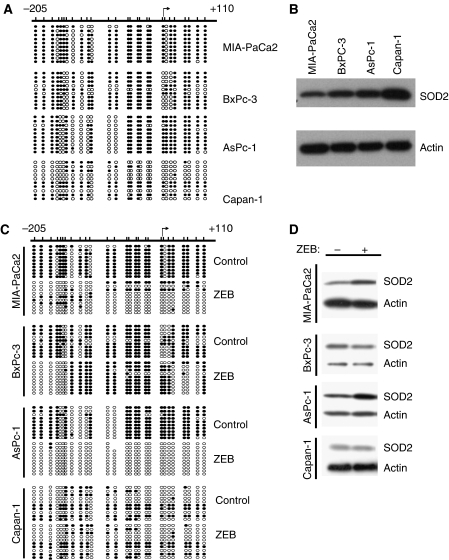
The *sod2* promoter is hypermethylated in some pancreatic carcinomas that is inversely correlated with protein levels. (**A**) The methylation status of the *sod2* promoter was examined by sodium bisulphite sequencing in four pancreatic carcinomas. All CpG dinucleotides are represented by either an open circle (○) to indicate unmethylated cytosines or a filled circle (•) to indicate a methylated cytosine. (**B**) Protein levels of SOD2 were determined by western blot. The level of SOD2 expression is inversely correlated with the extent of hypermethylation, with Capan-1 showing the lowest level of methylation and the highest level of expression. Actin serves as a loading control. (**C**) Sodium bisulphite sequencing of three cell lines that showed hypermethylation of the *sod2* promoter following treatment with a methyltransferase inhibitor, zebularine, for 72 h. (**D**) Protein expression levels of SOD2 in pancreatic carcinoma lines following zebularine treatment.

Several reports have demonstrated decreased expression of SOD2 in pancreatic carcinoma (Su *et al*, 2002; Cullen *et al*, 2003) without determining the underlying cause. To determine if decreased SOD2 expression could be the result of hypermethylation of the promoter, we performed sodium bisulphite sequencing on four pancreatic cell lines. We observed a high methylation density of the *sod2* promoter in MIA-PaCa2, intermediate methylation densities in AsPc-1 and BxPc-3, and a low density in Capan-1 (Figure 1A). The degree of methylation was inversely correlated with protein expression levels (Figure 1B).

### Methylation of the *sod2* promoter can be reversed with zebularine resulting in re-expression of SOD2

To further substantiate the finding of hypermethylation of the *sod2* promoter, we treated the three cell lines with zebularine (Driscoll *et al*, 1991), a methyltransferase inhibitor (Zhou *et al*, 2002), and determined methylation by sodium bisulphite sequencing (Figure 1C). Treatment with zebularine nearly eliminated methylation of the *sod2* promoter in both MIA-PaCa2 and AsPc-1. In BxPc-3, methylation was minimally decreased and showed a more limited pattern of demethylation with three regions showing a greater extent of demethylation than the remaining areas. Capan-1 showed no significant changes in methylation densities. To determine if the demethylation in response to zebularine treatment lead to a corresponding increase in expression of SOD2, we performed a western blot following zebularine treatment. Both MIA-PaCa2 and AsPc-1 showed a significant increase in SOD2 protein levels (Figure 1D). There was no increase in SOD2 expression in both BxPc-3 and Capan-1 upon treatment with zebularine (Figure 1D) consistent with the lesser extent of demethylation.

### SOD2 expression levels predict 2ME2 sensitivity that can be ameliorated by introduction of SOD2

**Figure 2:**
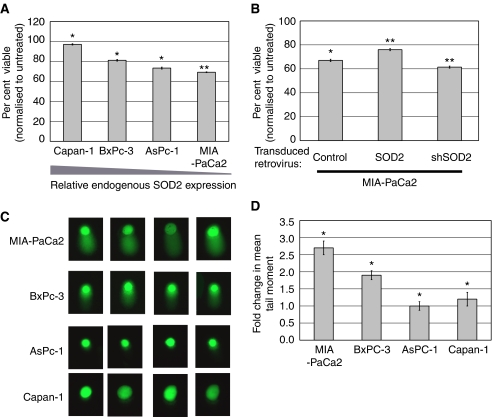
SOD2 protein levels modulate sensitivity to 2-methoxyestradiol and DNA damage. (**A**) Sensitivity to 2ME2 is inversely correlated with SOD2 expression levels. Following treatment with 3 *μ*M 2ME2 for 48 h, an MTT assay was performed in triplicate. The number of viable cells following 2ME2 treatment is shown as a per cent of the number of viable of vehicle-treated cells. ^*^P⩽0.05, ^**^P<0.001. A representative of three experiments is shown. (**B**) Determination of sensitivity to 2ME2 was performed on MIA-PaCa2 cell lines with engineered SOD2 levels in triplicate by MTT. A representative of three experiments is shown. The number of viable cells following 2ME2 treatment is shown as a per cent of the number of viable of vehicle-treated cells. Increased expression of SOD2 results in decreased sensitivity, whereas decreased SOD2 levels result in increased sensitivity. ^*^P⩽0.05, ^**^P<0.001. (**C**) DNA damage was determined by comet assay following treatment with 2ME2. Four representative nuclei for each cell lines are shown. (**D**) The extent of DNA damage following 2ME2 was determined by measuring the length of the tail moment of 50 nuclei. The fold-over-untreated mean tail moment length is shown where an asterisk indicates a *P*-value ⩽0.05.

**Figure 3:**
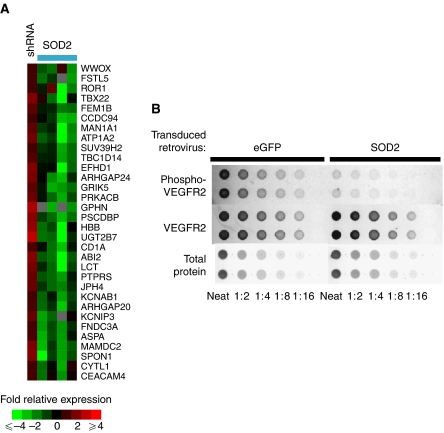
SOD2 expression in MIA-PaCa2 cells results in downregulation of several genes and phosphorylation of VEGFR2. (**A**) Oligonucleotide arrays were performed on SOD2-engineered MIA-PaCa2 cell lines. All named genes showing at least a two-fold downregulation in two of three SOD2 overexpression experiments as well as an increase in the shSOD2 experiment are shown. (**B**) Reverse-phase protein arrays were performed to determine differences in the phosphoproteome following manipulation of SOD2 protein levels. Phosphorylation of VEGFR2 is decreased in SOD2-overexpressed MIA-PaCa2 cells. Total VEGFR2 expression remains unchanged between the vector-only control and the SOD2-overexpressed cells, with total protein shown as the loading control.

Pancreatic carcinomas have been shown to be sensitive to 2ME2 (Schumacher *et al*, 1999; Qanungo *et al*, 2002). The mechanism of 2ME2-induced apoptosis involves mitochondrial-dependent pathways (Qanungo *et al*, 2002) as well as ROS (Chauhan *et al*, 2003). With diminished capacity to detoxify superoxide radicals, cells with silenced SOD2 should exhibit higher sensitivity to 2ME2. Using the four pancreatic carcinoma cell lines with variable SOD2 expression, we examined 2ME2 sensitivity in an MTT assay (Figure 3A). Capan-1, which has the highest endogenous expression of SOD2, was the least sensitive to 2ME2, whereas MIA-PaCa2, which has the lowest endogenous expression of SOD2, showed the highest sensitivity to 2ME2 (Figure 2A). MIA-PaCa2 cells are sensitive to 2ME2 (Schumacher *et al*, 1999), and the inhibition of MIA-PaCa2 measured in the MTT assay is similar to the 42% of apoptotic nuclei in MIA-PaCa2 following 2ME2 treatment for 48 h reported by Qanungo *et al* (2002). To test whether SOD2 expression itself could decrease sensitivity to 2ME2, we engineered MIA-PaCa2 cells with altered levels of SOD2. We demonstrate that these cell lines show both the expected level of expression and enzymatic activity (Supplementary Figure 1). SOD2 overexpression in MIA-PaCa2 leads to a slight reduction in sensitivity to 2ME2, whereas SOD2 knockdown showed a slight increase in sensitivity (Figure 2B).

### Treatment with 2ME2 leads to DNA damage and apoptosis of pancreatic cell lines

Since 2ME2 induces ROS and PARP activation in pancreatic cell lines (Qanungo *et al*, 2002), we tested whether or not 2ME2 induced DNA damage. Following treatment with 2ME2, there was significant DNA damage induced in both MIA-PaCa2 and BxPc-3 (Figure 2C). MIA-PaCa2 showed a nearly three-fold increase in tail length following treatment with 2ME2 as compared to control (Figure 2C), whereas BxPc-3 showed an approximate two-fold increase in tail length following 2ME2 treatment (Figure 2C). Both AsPc-1 and Capan-1 showed little to no tailing following treatment with 2ME2, indicating that 2ME2 did not lead to DNA damage in these cell lines (Figure 2C and D). Capan-1 has the highest expression of SOD2 and therefore the largest capacity to clear superoxide radicals, which may account for the limited DNA damage by 2ME2. While BxPc-3 shows a modest level of SOD2 expression, there was no tailing in response to 2ME2, which may be due to increased expression of other ROS-scavenging enzymes or better DNA repair mechanisms. While these results suggest that SOD2 expression can reduce 2ME2-induced DNA damage, we sought to directly determine if SOD2 expression alone could ameliorate the effects of 2ME2. Therefore, we performed a comet assay on MIA-PaCa2 with and without overexpression of SOD2. The overexpression of SOD2 in MIA-PaCa2 substantially lessened the extent of DNA damage incurred during treatment with 2ME2, whereas treatment of the shSOD2 MIA-PaCa2 cells resulted in increased damage (data not shown).

### Gene expression changes result from the overexpression and knockdown of SOD2

To address the changes in gene expression that result from SOD2 overexpression, we performed oligonucleotide arrays on MIA-PaCa2 with SOD2 overexpression and knockdown. All genes showing at least a two-fold change in expression in three of the four overexpression experiments and an inverse change in the knockdown experiment are shown in Figure 3A. There were 33 genes that showed a decrease in expression in the overexpression experiments by the preceding criteria but no genes showing an increase in expression by these same criteria. These genes are involved in many different cellular processes, including calcium homeostasis (*fstl5*, *man1a1*, *efhd1*, *prkacb*, and *kcnip3*), adhesion (*pscdbp* and *spon1*), GTP metabolism (*tbc1d14* and *arhgap20*), and steroid metabolism (*wwox* and *ugt2b7*).

### Signalling pathways showing altered activity with SOD2 expression

ROS has been shown to alter phosphorylation states of various proteins, especially tyrosine phosphorylation. In order to investigate the role of ROS on the phosphoproteome, we performed RPAs with lysates from MIA-PaCa2 containing altered SOD2 levels. Using these arrays, we identified a decrease in the phosphorylation of VEGFR2 when SOD2 was overexpressed (Figure 3B). Other reports have shown modification of VEGFR2 in response to various ROS-inducing agents, including H_2_O_2_ (Gonzalez-Pacheco *et al*, 2006), which is presumably increased in SOD2-deficient cell lines. In contrast, there was no change in the phosphorylation status of EGFR, STAT3, c-abl, or p-src detected using our RPAs (Supplementary Table 1).

### Changes in transcription factor binding as a result of SOD2 expression

In recent years, there has been considerable evidence that ROS leads to changes in transcription factor binding. In order to determine transcription factors that have altered DNA binding as a result of SOD2 expression, we performed Panomic's Protein/DNA array that examines the binding activities of 354 transcription factor binding sites. The experiment was conducted using nuclear extracts from control MIA-PaCa2 cells, MIA-PaCa2 overexpressing SOD2, as well as MIA-PaCa2 with SOD2 knockdown, and transcription factors showing reciprocal changes in overexpression and SOD2 knockdown are summarised in Table 1. Consistent with the gene expression data, there were more transcription factors identified that showed a decrease in binding upon SOD2 overexpression. Among those showing the greatest decrease in binding activity were Brn-3, Myc/Mad, and TCF/LEF.

### Identification of cognate binding sites for regulated transcription factors in the promoters of regulated genes

With the identification of both transcription factors showing altered DNA binding upon SOD2 expression and genes regulated by SOD2, we sought to identify cognate binding sites for the regulated transcription factors in the promoters of the regulated genes. Of the 32 genes identified as being downregulated by SOD2 expression (Figure 3), promoter regions were found for 30 of them using Genomatix's Gene2Promoter database. The promoter regions were scanned for binding sites of the downregulated transcription factors listed in Table 2 using Genomatix's MatInspector. Of the 30 promoters searched, binding sites were identified in 25 of them. Table 2 summarises these results.

## DISCUSSION

The generation of ROS is a by-product of several cellular processes, including electron transport (Cadenas, 1989). The elimination of ROS is handled in the cell by several antioxidant enzymes, including superoxide dismutases, which convert the superoxide radical to H_2_O_2_. The H_2_O_2_ then gets converted into water and molecular oxygen by peroxidases. The perturbation of any of these processes can result in the build up of deleterious ROS in the cell. ROS is implicated in the pathology of several cancers, including pancreatic carcinoma (Cullen *et al*, 2003). Therefore, we sought to better understand the role of ROS in pancreatic carcinoma. In particular, we were interested in the effects that the manipulation of SOD2 expression levels has in pancreatic carcinoma cell lines, since they have decreased expression of SOD2 resulting in increased proliferation rates.

One mechanism employed by cells for decreasing gene expression levels is hypermethylation of the promoter. We have recently shown that the *sod2* promoter is hypermethylated in multiple myeloma (Hodge *et al*, 2005a), so we sought to determine if hypermethylation may be an explanation for the decreased expression of SOD2 observed in pancreatic carcinoma cell lines. Through sodium bisulphite sequencing, we show that the promoter of *sod2* is hypermethylated in several cell lines (Figure 1), including MIA-PaCa2. Furthermore, the expression of SOD2 was increased following treatment of the cells with the methyltransferase inhibitor zebularine. The role of hypermethylation in the silencing of *sod2* in primary pancreatic carcinoma needs to be determined, but our initial studies using the pancreatic carcinoma cell lines indicate a possible role for hypermethylation.

Decreased expression of SOD2 would, theoretically, render the cells less capable of eliminating ROS that may build up in the cell. The decreased ability of cancer cells to eliminate ROS represents an Achille's heel that can be exploited to induce apoptosis of these cells. Indeed, cells with lower expression of SOD2 are more sensitive to the oxidative burst agent, 2ME2 (Figure 2). Therefore, for carcinomas in which there is decreased SOD2 activity, regardless of the mechanism, treatment with 2ME2 or other agents resulting in oxidative bursts may represent a potential treatment option.

While lowered SOD2 expression in itself might present treatment options for pancreatic carcinoma, the furthered understanding of the consequences of altered SOD2 expression illuminates other mechanisms that could be involved in the pathology of pancreatic cancer, suggesting further treatments. To this end, we examined in an integrated proteomic and genomic manner the consequences of SOD2 expression in MIA-PaCa2 cells by both overexpression and knockdown of SOD2. Using this approach, we have identified the genes that are regulated by SOD2 as well as the transcription factors whose binding activities are altered in response to SOD2 expression. The genes identified in Figure 3 function in diverse cellular processes, including calcium binding, steroid metabolism, and cell adhesion. Calcium binding is the most represented function according to Gene Ontology terms and is affected by the expression of five out of the 32 genes shown in Figure 3 (*fstl5*, *man1a1, efhd1, prkacb*, and *kcnip3*). The regulation of calcium homeostasis is complex, but there is evidence that ROS and calcium signalling pathways interact (reviewed by Yan *et al*, 2006). The identification of the transcriptional regulation of several genes involved in calcium regulation suggests that there may be an even greater interplay between these two signalling molecules and bears further investigation.

We identified the transcription factors in MIA-PaCa2 that show altered binding activity in response to altered levels of SOD2 (Table 1). First, it is interesting to point out that nearly all of the transcription factors identified show decreased binding. Second, almost all of these transcription factors are also impacted by calcium signalling. While the genes that are regulated by SOD2 expression could be regulated in some other manner, we sought to determine if there was overlap in the transcription factors identified as having SOD2-altered binding activities and the genes identified as being SOD2-regulated. In order to do this, we first identified the potential promoters of these genes using Genomatix's Gene2Promoter database. Once identified we used Genomatix's MatInspector to determine the transcription factor binding modules. More than 80% of the promoters searched contained binding sites for at least one of the regulated transcription factors. The identification of binding sites for SOD2-regulated transcription factors in the promoters of regulated genes (Table 2) strengthens the use of computer analysis to begin to identify signalling networks that may be at play in the cells studied.

We have shown that altered levels of SOD2 reveal a sensitivity to oxidative stress created by 2ME2 treatment and result in perturbations in the binding activities of several transcription factors, decreased phosphorylation of VEGFR2, and the alteration of the expression of several genes. The ability to determine both the genomic and proteomic events that change as a result of SOD2 expression, and therefore the redox status of the cell, provides us with a wealth of complex information. We have begun to understand the molecular consequences of altered SOD2 activity in pancreatic cancer and, furthermore, we have determined that the lack of SOD2 expression may result in a therapeutic advantage.

## Figures and Tables

**Table 1:** Transcription factors with modulated DNA binding activities in SOD2-altered MIA-PaCa2 cells

**Transcription factor**	**Fold change SOD2**	**Fold change shSOD2**
*Transcription factors with decreased DNA binding upon infection with SOD2 retrovirus*		
Bm-3	−10.7	1.2
C/EBP	−7.5	3.6
NPAS2	−6.1	1.9
Myc-Max	−4.5	5.6
LyF-1	−3.7	2.0
TCF/LEF	−3.1	1.7
NRF-1	−2.8	2.6
WT1	−2.5	1.8
TCE	−2.1	4.6
LR1	−2.1	4.4
GATA-2	−1.8	2.0
SP1	−1.6	1.5

*Transcription factors with increased DNA binding upon infection with SOD2 retrovirus*		
Pax-6	2.3	−1.7
PCF	2.6	−1.7

SOD2=manganese superoxide dismutase.

Transcription factors with altered binding activity in response to engineered SOD2 expression levels in MIA-PaCa2 cells. Transcription factor binding activity was determined by Panomic's DNA/Protein Combo array that profiles the binding activity of 390 transcription factors. Transcription factors that showed reciprocal binding patterns with SOD2 overexpression and knockdown are shown.

**Table 2:** Identification of binding sites in the promoters of SOD2-regulated genes for transcription factors showing altered DNA binding in MIA-PaCa2 cells with modulated SOD2

**Transcription factor module**	**Gene promoters containing modules**
AP1_CEBP	*wwox*
CEBP_STAT	*wwox, fem1b*
SP1_ETS	*wwox*, *tbx22*, *man1a1*, *suv39h2*, *tbc1d14*, *efhd1*, *arhgap24*, *grik5*, *prkacb, gphn*, *abi2*, *jph4 (ap1g2)*, *kcnab1*, *kcnip3 (csen)*, *fndc3a*, *aspa*, *mamdc2*
ETS_CEBP	*atp1a2*, *prkacb*, *fndc3a*
EGR_SP1	*fstl5*, *fem1b*, *man1a1*, *tbc1d14*, *efhd1*, *arghap24*, *prkacb*, *abi2*, *jph4 (ap1g2), kcnip3 (csen), fndc3a*
CAAT_SP1	*ror1*, *man1a1*
CEBP_SP1	*man1a1*, *prkacb*, *kcnab1*
BRN_p53	*prkacb, ugt2b7*
BRN_RORA	*kcnab1*, *kcnip3 (csen)*
CP2_SP1	*hbb, ceacam4*
SP1_CREB	*spon1*

SOD2=manganese superoxide dismutase.

Binding sites for SOD2-regulated transcription factors in the promoters of SOD2-regulated genes. The promoters of the genes determined to be regulated by SOD2 (Figure 3) were identified using Genomatix's Gene2Promoter database (www.genomatix.de). Once identified, the promoters were mapped for transcription factor binding sites using MatInspector (Genomatix, Ann Arbor, MI, USA). This table shows all the SOD2-regulated transcription factor (listed in this table) binding sites in the promoters of the SOD2-regulated genes.
