# Supplementary material for: Molecular consequences of SOD2 expression in epigenetically silenced pancreatic carcinoma cell lines
Source: Br J Cancer. 2007 Sep 25;97(8):1116–23. doi: 10.1038/sj.bjc.6604000 (PMC2360443; doi:10.1038/sj.bjc.6604000)
Supplement: Supplementary data [file 6604000x1.doc]

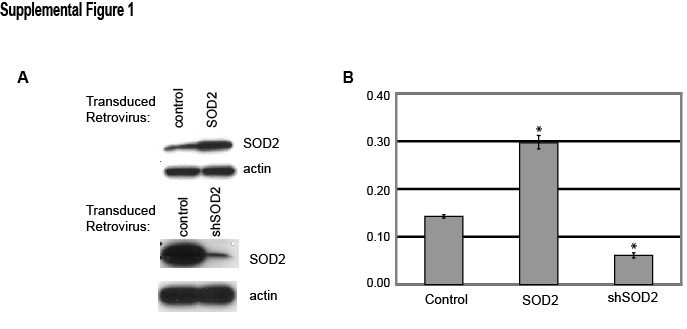


**Supplemental Figure 1**  **The generation of MIA-PaCa2 stable cell lines with altered SOD2 expression and enzymatic activity.** The MIA-PaCa2 cells were infected with viral supernatant from amphotropic Pheonix cells transfected with either an eGFP control or with a SOD2-eGFP vector. Forty-eight hours later cells were sorted for GFP expression and were >98% pure. For knockdown experiments, the MIA-PaCa2 cell line was infected with viral supernatant from amphotropic Pheonix cells transfected with either empty pRetroSuper or shSOD2-pRetroSuper. Cells were selected with 1µg/ml puromycin. **A.,** Western blots were performed using 10 µg protein, with actin as a loading control. The shSOD2 blot was overexposed to show the light SOD2 band. **B.,** Enzymatic activity of MIA-PaCa2 cell lines with altered SOD2 levels was determined. An asterisk indicates a p-value < 0.001. There is a 3-fold increase in SOD2 protein expression and a 2-fold increase in enzymatic activity. For the knock-down of SOD2 there is a 95% reduction in expression with a 2-fold decrease in enzymatic activity.

**Supplemental Table 1. Reverse-phase protein arrays.**

| **Antibodies** | **Fold Change** | **Antibody Source** |
| --- | --- | --- |
| JAK1/ phospho-JAK1 (Tyr1022/1023) | 1.0 | Biosource #44-400G/ Cell Signaling #3331S |
| IkB/ phospho-IkB (Ser32) | 1.0 | Cell Signaling #9242/ Cell Signaling #9241 |
| STAT5A/ phospho-STAT5 (Tyr694) | 1.2 | BD Pharmingen #611834/ Cell Signaling #9351S |
| STAT5B/ phospho-STAT5 (Tyr694) | 1.3 | BD Pharmingen #556517. Cell Signaling #9351S |
| c-abl/ phospho-c-abl (Thr735) | 1.0 | Upstate #06-465/ Cell Signaling #2864S |
| STAT3/ phospho-STAT3 (Tyr705) | 1.0 | Upstate #06-596/ Cell Signaling #9131S |
| EGFR/phospho-EGFR (Tyr1068) | 1.0 | Sigma #E3138/Cell Signaling #2234S |
| VEGFR2/phospho-VEGFR2 (Tyr996) | 3.2 | Cell signaling # 2479/Cell Signaling 2474 |
| phospho-Tyr | 1.3 | Upstate # 05-321 |
| phospho-src (Tyr416) | 1.6 | Cell Signaling 2101S |

The normalized results for all tested antibodies are presented. The only significant change found was for the phosphorylation of VEGFR2. The sources for all antibodies are also listed.
